# Abnormal Seebeck Effect in Vertically Stacked 2D/2D PtSe_2_/PtSe_2_ Homostructure

**DOI:** 10.1002/advs.202203455

**Published:** 2022-11-10

**Authors:** Won‐Yong Lee, Min‐Sung Kang, Jae Won Choi, Si‐Hoo Kim, No‐Won Park, Gil‐Sung Kim, Yun‐Ho Kim, Eiji Saitoh, Young‐Gui Yoon, Sang‐Kwon Lee

**Affiliations:** ^1^ Department of Physics Center for Berry Curvature based New Phenomena Chung‐Ang University Seoul 06974 Republic of Korea; ^2^ Division of Solid State Electronics Department of Electrical Engineering Uppsala University Uppsala 75103 Sweden; ^3^ Department of Applied Physics The University of Tokyo Tokyo 113–8656 Japan

**Keywords:** homostructure, hot‐carrier injection, in‐plane Seebeck effect, interfacial Seebeck effect, Mott relation, platinum diselenide, van der Waals

## Abstract

When a thermoelectric (TE) material is deposited with a secondary TE material, the total Seebeck coefficient of the stacked layer is generally represented by a parallel conductor model. Accordingly, when TE material layers of the same thickness are stacked vertically, the total Seebeck coefficient in the transverse direction may change in a single layer. Here, an abnormal Seebeck effect in a stacked two‐dimensional (2D) PtSe_2_/PtSe_2_ homostructure film, i.e., an extra in‐plane Seebeck voltage is produced by wet‐transfer stacking at the interface between the PtSe_2_ layers under a transverse temperature gradient is reported. This abnormal Seebeck effect is referred to as the interfacial Seebeck effect in stacked PtSe_2_/PtSe_2_ homostructures. This effect is attributed to the carrier‐interface interaction, and has independent characteristics in relation to carrier concentration. It is confirmed that the in‐plane Seebeck coefficient increases as the number of stacked PtSe_2_ layers increase and observed a high Seebeck coefficient exceeding ≈188 µV K^−1^ at 300 K in a four‐layer‐stacked PtSe_2_/PtSe_2_ homostructure.

## Graphical abstract

A novel strategy to increase the Seebeck coefficient of 2D PtSe_2_ films by stacking the same PtSe_2_ layer onto each other as stacked PtSe_2_/PtSe_2_ homostructures via a wet‐transfer method is demonstrated. The Seebeck coefficient increases significantly with increasing number of stacking films in a PtSe_2_/PtSe_2_ homostructure for a four‐stacked homostructure due to the carrier‐interface interaction under the longitudinal temperature gradient.

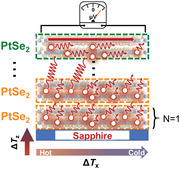

## 1 Introduction

Over the past decades, thermoelectric (TE) materials have been extensively studied due to their potential use in energy harvesting applications.^[^ ^1^, ^2^, ^3^, ^4^, ^5^, ^6^, ^7^, ^8^ ^]^ The efficiency of TE materials depends on the dimensionless figure of merit, *ZT* = *S* ^2^ *σT*/*κ*, where *σ* is the electrical conductivity, *S* is the Seebeck coefficient, *T* is the absolute temperature, and *κ* is the thermal conductivity.^[^ ^4^, ^9^, ^10^, ^11^, ^12^ ^]^ Hence, a high‐performance TE material should have a high power factor (*S^2^σ*) and lower thermal conductivity. Therefore, two‐dimensional (2D) metal dichalcogenides (TMDC) are promising materials for TE applications due to their superior electronic and phonon transport properties, provided by their ideal high‐performance TE structural features, i.e., large energy bandgaps and atomically thin layers.^[^ ^13^, ^14^, ^15^ ^]^ Particularly, platinum diselenide (PtSe_2_) has been proven to be a promising candidate as an excellent TE material because of its tunable energy bandgap and high mobility.^[^ ^14^, ^16^, ^17^, ^18^, ^19^ ^]^ Generally, its electronic structure is highly sensitive to its thickness. For example, an untrained single‐crystalline monolayer PtSe_2_ is a semiconductor with an energy band gap of ≈1.2 eV, and it exhibits semi‐metallic behavior when the thickness is increased to three layers or more.^[^ ^14^, ^16^ ^]^ Recently, Moon et al. reported that they observed an extremely high Seebeck coefficient (>1 mV K^−1^) in a bilayer of single‐crystalline PtSe_2_ with the gate voltage sweeping from −5 to 80 V at 300 K.^[^ ^14^ ^]^ Nevertheless, the overall power factor and TE performance of this material are not sufficient. In addition, systematic studies of a large‐area millimeter‐scale PtSe_2_ layer offering throughputs that can meet the practical application demands remain challenging.

Moreover, various studies have been conducted on artificial 2D van der Waals (vdW) junctions using homogeneous or heterogeneous 2D TMDC materials due to the diversity and considerably wide coverage properties of these materials. For instance, Prospischil and colleagues studied the formation of 2D homojunction using monolayer WSe_2_ layers^[^ ^20^ ^]^ via electrostatic doping in the same WSe_2_ monolayer‐based p‐n junction diodes. However, there have been no reported studies on TE properties, including the Seebeck coefficient and electrical conductivity, using 2D TMDC materials as a form of the homojunction structure (i.e., 2D/2D TMDC homostructure). Elucidation of the underlying mechanism governing the interface effect in the transferred 2D/2D TMDC homostructure, integrating more than one type or one more 2D layer onto a single substrate, remains challenging. The Seebeck coefficient of a conducting solid, including semiconductors, is induced by the asymmetry of the electronic density of states (DOS) and the carrier mobility at the Fermi‐level energy (*E*_F_) defined by the Mott relation, which can be expressed as follows^[^ ^21^ ^]^:

(1) $$S=\frac{\pi^{3}}{3}\left(\frac{k_{B}^{2}T}{q}\right){\left\{\frac{1}{N}\frac{dN\left(E\right)}{dE}+\frac{1}{\mu }\frac{d\mu \left(E\right)}{dE}\right\}}_{E=E_{F}}$$

where *k_B_* is the Boltzmann constant, *q* is the carrier charge, *N* is the carrier concentration, *μ* is the carrier mobility, and *E*_F_ is the Fermi energy. In the 2D/2D homostructure, the total Seebeck coefficient (*S*_T_) of the stacked 2D/2D homostructure can be expressed by the weighted average, which is given by

(2) $$S_{T}=\frac{\sum_{i}S_{i}\sigma_{i}}{\sum_{i}\sigma_{i}}$$

where *S*_i_ and *σ*_i_ are the Seebeck coefficient and the electrical conductance of each 2D layer, respectively. According to Equations (1) and (2), the total Seebeck coefficient is equal to that of a single 2D structure when one or more 2D materials are stacked on the same 2D materials onto a single substrate in the form of 2D/2D/⋅⋅⋅/2D homostructure.

Here, we report a novel strategy to increase the intrinsic Seebeck coefficient of multilayer PtSe_2_ films by stacking the same PtSe_2_ layer onto each other as stacked PtSe_2_/PtSe_2_ homostructures via a wet‐transfer method. We observed that the Seebeck coefficient increases significantly with an increasing number of stacking layers in a PtSe_2_/PtSe_2_ homostructure, and exceeds 188 µV K^−1^ for a four‐layer‐stacked homostructure, i.e., ≈260% enhancement compared to that for a single PtSe_2_ film (≈72 µV K^−1^), at 300 K. This unusual behavior of the Seebeck coefficient can be explained by the extra interfacial Seebeck effect at the interface between the PtSe_2_ layers mainly because of the longitudinal temperature gradient of the sample. We also discuss the role of the upper and lower layers in terms of the Seebeck coefficient and TE power factor in stacked PtSe_2_/PtSe_2_ homostructures, along with the dependence of layer thickness.

## 2 Results and Discussion

**Figure 1:**
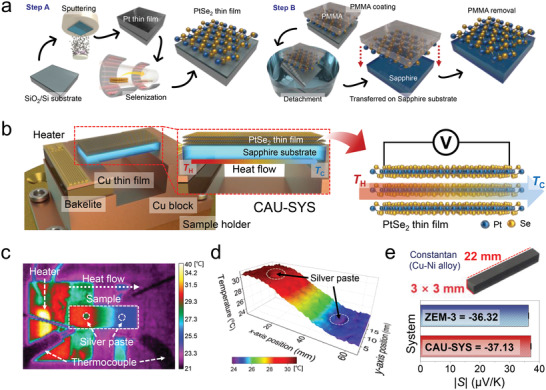
Fabrication and measurement of in‐plane TE property of the PtSe_2_ thin films. a) Fabrication process and atomic structures of PtSe_2_ thin films on the SiO_2_/Si substrate using direct selenization chemical vapor deposition (CVD). Each Pt thin film was deposited onto SiO_2_/Si substrate and then converted into the PtSe_2_ thin film via selenization (Step A). The grown PtSe_2_ thin film was transferred onto sapphire substrate using a wet‐transfer process (Step B). To remove the substrate effect, we adopted the sapphire substrate when measuring TE properties using our measurement system. By repeating the wet‐transfer process, we formed the PtSe_2_ homostructure. b) In‐house measurement system (CAU‐SYS) used to determine in‐plane Seebeck coefficient (*S*_||_) and electrical conductivity (*σ*_||_) for the single PtSe_2_ films and stacked PtSe_2_/PtSe_2_ homostructures. Enlarged image shows the structure of the sample and the heat flow along the sample. *T*_H_ and *T*_c_ refer to hot and cold regions, respectively. c) Infrared thermal image (top surface view) of the stacked PtSe_2_/PtSe_2_ homostructure during the measurement in the vacuum chamber. d) Temperature profile achieved from the infrared thermal image between two points (*T*_H_ and *T*_C_ in Figure 1b) along the sample (transverse direction). e) The *S*_||_ of the constantan sample (width × depth × length = 3 mm × 3 mm × 22 mm) measured using the commercial system (ZEM‐3) and our in‐house CAU‐SYS, showing identical values with acceptable reliability.

We first deposited Pt layers on a SiO_2_/Si substrate of thickness ≈270 nm by a sputtering process, and then converted those into millimeter‐scale PtSe_2_ thin films via selenization (Step A in **Figure** **1**a). Since the PtSe_2_ thin films were fabricated by the selenization process, we adjusted the thickness of the PtSe_2_ thin films from monolayer to multilayer by altering the Pt deposition thickness. For instance, we synthesized ≈3 nm thick 2D PtSe_2_ films using simple selenization of pre‐deposited Pt thin films (thickness of ≈0.5 nm). Subsequently, the deposited PtSe_2_ thin film was transferred onto the sapphire substrate via the wet‐transfer process (Step B in Figure 1a). In this step, we formed the sample structure required for determining in‐plane TE properties (Seebeck coefficient (*S*_||_) and electrical conductivity (*σ*_||_)) using our measurement system (CAU‐SYS) and fabricated the vertically stacked PtSe_2_/PtSe_2_ homostructure by sequential repetition of this step. All samples were measured on sapphire (Al_2_O_3_) substrate to avoid substrate effects during Seebeck coefficient measurement,^[^ ^22^ ^]^ and measurement errors were estimated to be ≤2%. The details regarding the fabrication of the 2D PtSe_2_ thin films are given in our previous literature.^[^ ^23^, ^24^ ^]^ With the in‐house CAU‐SYS for measuring Seebeck coefficient of 2D PtSe_2_ films, we confirmed that the heat flow is well formed along the in‐plane direction using an infrared thermal camera (Figure 1c) and the in‐plane Δ*T*_||_ of the PtSe_2_ thin films is properly maintained at 5 K (Figure 1d). In addition, to validate the reliability of the in‐house CAU‐SYS, we conducted the same measurement on constantan alloy (Cu55–Ni45 wt.%, 3 mm × 3 mm × 22 mm) as a reference sample using a commercial equipment (ZEM‐3, ULVAC Riko, Inc.) at 300 K (Figure 1e), confirming that the CAU‐SYS achieved satisfactory reliability.

**Figure 2:**
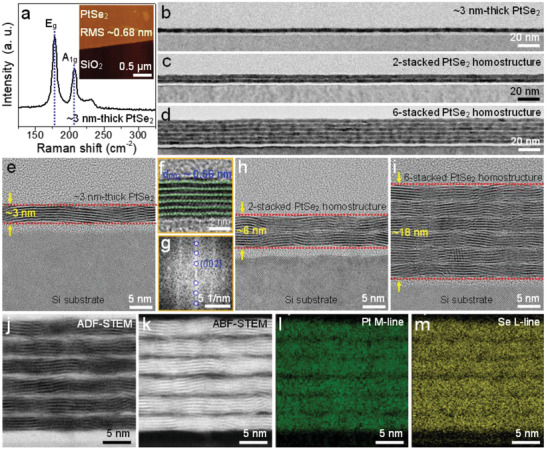
Structural and morphological analyses of as‐grown and vertically stacked PtSe_2_ thin films. a) Raman spectrum of ≈3 nm thick PtSe_2_ thin film on the SiO_2_/Si substrate. Inset shows the AFM image of the as‐grown PtSe_2_ film. b–d) Cross‐sectional HR‐TEM images of one‐, two‐, and six‐stacked PtSe_2_/PtSe_2_ homostructures prepared by a wet‐transfer method. e–g) Enlarged HR‐TEM images and corresponding FFT pattern of the ≈3 nm thick PtSe_2_ thin film. h,i) Enlarged HR‐TEM images of two‐ and six‐stacked PtSe_2_/PtSe_2_ homostructures. j–m) ADF‐ and ABF‐STEM images of the six‐stacked PtSe_2_/PtSe_2_ homostructure and the corresponding elemental mappings of Pt (M‐line, green) and Se (L‐line, yellow).

As‐grown PtSe_2_ thin film on the SiO_2_/Si substrate exhibits two prominent peaks at ≈178 and ≈207 cm^−1^ in the Raman spectrum (**Figure** **2**a), which can be assigned to the in‐plane (*E*_g_) and out‐of‐plane (*A*_1g_) modes,^[^ ^25^, ^26^, ^27^ ^]^ respectively. The atomic force microscopy (AFM) image (inset image in Figure 2a) shows that the PtSe_2_ thin film has good surface roughness with root mean square (RMS) < 1 nm. Cross‐sectional transmission electron microscopy (TEM) specimens were prepared using the focused ion beam milling method to further investigate the crystallographic characteristics of the vertically stacked PtSe_2_ films. The representative cross‐sectional TEM images of single, two‐, and six‐stacked PtSe_2_ films (Figures 2b–d) indicate large‐area continuous stacking morphologies and apparent interface formations between adjacent vertically stacked PtSe_2_ films. From the enlarged high‐resolution TEM (HR‐TEM) images and corresponding fast Fourier transform (Figures 2e–g), the ≈3 nm thick PtSe_2_ film exhibits a horizontally aligned vdW structure consisting of six layers. The interplanar d‐spacing value is determined to be ≈0.56 nm, which is assigned to the (001) interplanar distance of the PtSe_2_ octahedral 1T phase.^[^ ^26^, ^28^ ^]^ Through Raman and TEM analyses, we confirmed the complete conversion of the ≈0.5 nm thick Pt film into the ≈3 nm thick PtSe_2_ thin film. Moreover, we also observed that the 3 nm thick PtSe_2_ thin film exhibits homogeneous morphology consisting of a large number of randomly oriented nanograins (lateral size < 30 nm). The enlarged HR‐TEM images of two‐ and six‐layer‐stacked PtSe_2_/PtSe_2_ homostructures (Figure 2h,i) show that vdW physical gaps are retained across the entire stacked PtSe_2_/PtSe_2_ homostructures, whereas different Z‐contrast is observed in the interfacial regions between adjacent vertically stacked ≈3 nm thick PtSe_2_ films incorporating identical morphological and crystallographic features. We performed cross‐sectional scanning TEM (STEM) analysis with energy‐dispersive X‐ray (EDX) mapping to further identify the interface of the six‐stacked PtSe_2_/PtSe_2_ homostructures. Figure 2j–m shows the ADF‐ and ABF‐STEM images and corresponding elemental mappings of Pt (M‐line, green) and Se (L‐line, yellow), respectively. The Pt and Se distributions are consistent with the thickness of the vertically stacked PtSe_2_ films, whereas the deterioration of (001) lattice planes and low Pt and Se contents are observed at the interface between adjacent vertically stacked ≈3 nm thick PtSe_2_ films. These phenomena are associated with the O_2_ plasma treatment of an underneath PtSe_2_ thin film for fabricating wrinkle‐free and good adhesive vertically stacked PtSe_2_ homostructures. According to our previous report,^[^ ^23^ ^]^ Pt‐ and Se‐deficient atomic vacancies were formed on the top of O_2_ plasma‐treated PtSe_2_ thin films, thus forming interfaces in vertically stacked PtSe_2_/PtSe_2_ homostructures. From these observations, we note that the lattice orientation could not be physically controlled when stacking another layer of PtSe_2_ to prepare the homostructure, because the interface formation is related to the random stacking orientations caused by the polycrystalline nature of the synthesized PtSe_2_ films and structural deteriorations including Pt‐ and Se‐deficient atomic vacancies on the top of O_2_ plasma‐treated PtSe_2_ films.

**Figure 3:**
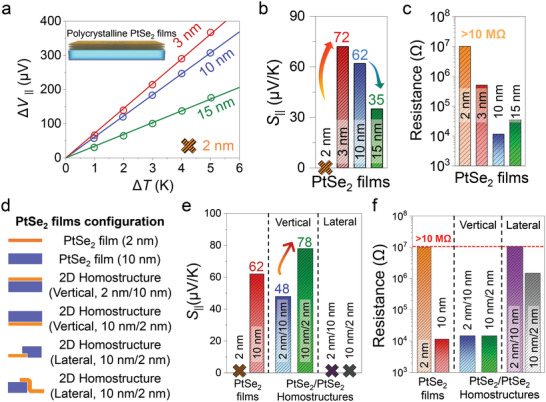
TE properties of PtSe_2_ thin films and vertically and laterally stacked PtSe_2_/PtSe_2_ bilayer homostructures. a) In‐plane TE voltage (Δ*V*_||_), b) Seebeck coefficient (*S*_||_), and c) electrical resistance (*R*) of PtSe_2_ thin films with the thicknesses of 2, 3, 10, and 15 nm, respectively. d) Schematics for PtSe_2_ thin films and vertically and laterally stacked PtSe_2_/PtSe_2_ bilayer homostructures. Comparison of the in‐plane e) Seebeck coefficient (*S*_||_) and f) resistance (*R*) for PtSe_2_ thin films and vertically and laterally stacked PtSe_2_/PtSe_2_ bilayer homostructures. The “X” mark indicates that it cannot be measured since the *R* of the sample is out of the measurement range.

The formation of the interface in the stacked PtSe_2_/PtSe_2_ homostructures affects not only the morphology but also the TE properties. To compare the TE properties between the PtSe_2_ films and the stacked PtSe_2_/PtSe_2_ homostructures, we first measured the TE properties of the PtSe_2_ thin films of various thicknesses (2, 3, 10, and 15 nm) (**Figure** **3**a–c). It could not measure the potential difference for the ≈2 nm thick PtSe_2_ thin film due to its extremely high electrical resistance (>10 MΩ). On the other hand, the measured *S*_||_ for 3‐, 10‐, and 15‐nm‐thick PtSe_2_ films were determined to be +72, +62, and +35 µV K^−1^, respectively, where the positive sign indicates a p‐type conduction in PtSe_2_ films, at 300 K, showing that the measured *S*_||_ values for the PtSe_2_ films tends to decrease as the thickness increases up to 15 nm (Figure 3b). This result is attributed to the dependence of semiconductor‐to‐semimetal transition on increasing the thickness of the PtSe_2_ materials. For instance, we confirmed the semi‐metallic behavior of PtSe_2_ thin films (≈6 nm)^[^ ^23^, ^24^ ^]^ by measuring the relationship between the electrical conductivity and temperature, corresponding well with the theoretical calculation. The theoretical calculation shows that a monolayer PtSe_2_ is a semiconductor with a band gap up to ≈1.2 eV, and the band gap of the PtSe_2_ rapidly reduces and becomes zero from the four‐layer PtSe_2_.^[^ ^29^, ^30^ ^]^ As shown in Figure 3c, we observed that the *R* of the ≈10 nm thick PtSe_2_ thin film (≈10 kΩ) was much lower than that of the ≈3‐nm‐thick PtSe_2_ thin film (≈500 kΩ). In addition, the in‐plane electrical conductivity and power factor of the single PtSe_2_ thin films were determined (Figure S1, Supporting Information).

In our previous study, we fabricated various kinds of vertically stacked heterostructures using PtX_2_ (*X* = S, Se, and Te) thin films and measured their in‐plane TE properties. As a result, we confirmed that which material was used for the top and bottom layers lead to very different TE properties.^[^ ^23^ ^]^ Moreover, to ensure the role of the interface and upper‐layer coverage in PtSe_2_/PtSe_2_ homostructures, we constructed four‐types of vertically and laterally stacked PtSe_2_/PtSe_2_ homostructures, which consisted of the 10 nm and 2 nm thick PtSe_2_ thin films (Figure 3d and Figure S2, Supporting Information). For instance, the vertically stackedPtSe_2_/PtSe_2_ homostructure (10 nm/2 nm) is made up of the 10 nm thick upper layer and the 2 nm thick lower layer, respectively. Compared with the *S*_||_ of the 10 nm thick PtSe_2_ thin film (≈62 µV/K), the *S*_||_ values of the vertically stacked PtSe_2_/PtSe_2_ homostructure (10 nm/2 nm) increased to ≈78 µV K^−1^ while the *S*_||_ of the vertically stacked PtSe_2_ bilayer (2 nm/10 nm) decreased to ≈48 µV K^−1^ (Figure 3e). On the other hand, the *R* values for these samples remain the same regardless of the stacking order (Figure 3f), indicating that the semi‐metallic 10 nm thick PtSe_2_ layer acts as the effective conducting path in vertically stacked PtSe_2_/PtSe_2_ bilayers (Figure 3e). Simultaneously, we also measured the TE properties for two laterally stacked PtSe_2_/PtSe_2_ homostructures (2 nm/10 nm and 10 nm/2 nm as seen in Figure 3d) to further investigate the carrier transport in the PtSe_2_/PtSe_2_ homostructures (Figures 3d−f). No *S*_||_ was obtained for both samples (Figure 3e,f) because it is difficult to occur the in‐plane carrier transport in the 2 nm thick PtSe_2_ thin film due to the high resistance (>10 MΩ) as shown in Figure 3c. From these results, we concluded that the carrier transport in the vertically stacked PtSe_2_/PtSe_2_ homostructure (2 nm/10 nm) was hindered since the 2‐nm‐thick PtSe_2_ thin film was used as the top layer. On the other hand, the *S*_||_ of the vertically stacked PtSe_2_/PtSe_2_ homostructure (10 nm/2 nm) was enhanced by the additional Seebeck effect, i.e., interfacial Seebeck effect (*S*_int_) occurred at the interface between the PtSe_2_ films, which was added to the *S*_||_ value of the 10 nm thick PtSe_2_ thin film.^[^ ^23^ ^]^ This effect appears clearly when comparing the *S*_||_ of the PtSe_2_ films with the same thickness (Figure S3, Supporting Information). Although the PtSe_2_ thin film (6 nm) and vertically stacked PtSe_2_/PtSe_2_ homostructure (3 nm/3 nm) are the same thickness, the *S*_||_ of the PtSe_2_/PtSe_2_ homostructure (3 nm/3 nm) is ≈170% larger than that of the PtSe_2_ thin film (6 nm) due to the interface as shown in Figure 2h. The mechanism associated with this phenomenon will be explained in more detail later.

**Figure 4:**
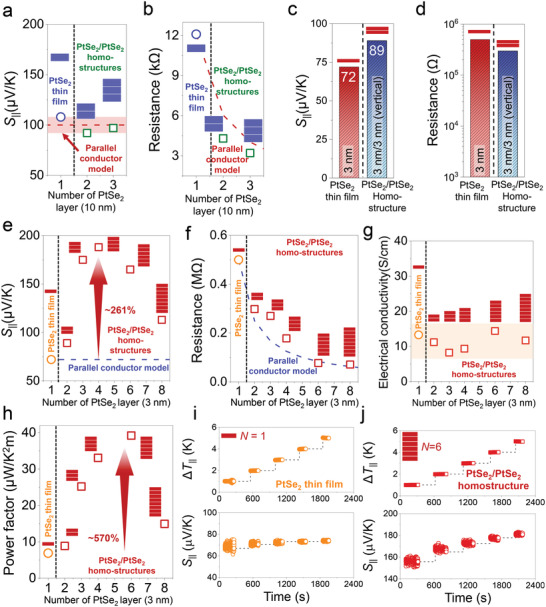
In‐plane TE properties for the alternatingly stacked PtSe_2_/PtSe_2_ homostructures. In‐plane a) Seebeck coefficients (*S*_||_) and b) resistances (*R*) for the one‐, two‐ and three‐stacked PtSe_2_/PtSe_2_ homostructure, where the 10 nm thick‐PtSe_2_ layer was used in PtSe_2_ homostructure. In‐plane c) Seebeck coefficient and d) resistance for the 3 nm thick PtSe_2_ thin film and the PtSe_2_/PtSe_2_ homostructure (3 nm/3 nm) on the sapphire substrate, respectively. In‐plane e) Seebeck coefficient, f) resistance, g) electrical conductivity, and h) power factor of the stacked PtSe_2_/PtSe_2_ homostructure as a function of the number of 3 nm PtSe_2_ film up to eight. Measured temperature difference and obtained in‐plane Seebeck coefficient for the i) 3 nm thick PtSe_2_ thin film (*N* = 1) and the j) vertically stacked PtSe_2_/PtSe_2_ homostructure (*N* = 6).

To further investigate on the *S*_int_ in the PtSe_2_/PtSe_2_ homostructure, we conducted the same experiments to measure the *S*_||_ of repeatedly stacked PtSe_2_ layers with the same thickness (**Figure** **4**a–f). As discussed in Equation (2), irrespective of the number of PtSe_2_ layers stacked in the PtSe_2_/PtSe_2_ homostructures, the total Seebeck coefficient in vertically stacked PtSe_2_/PtSe_2_ homostructures should be equal to that of a single PtSe_2_ film, according to the parallel conduction model. In Figure 4a, the measured *S*_||_ of alternatingly stacked PtSe_2_ layers with a thickness of 10 nm as a form of the vertically stacked PtSe_2_/PtSe_2_ homostructures remains unchanged as the number of PtSe_2_ layers was increased up to three (*N* = 1 – 3), and *R* decreases according to Ohm's law (Figure 4b), as we expected in the parallel conduction model. These results are attributed to the semi‐metallic property of the 10 nm thick PtSe_2_ thin film and the carrier mean free path in the PtSe_2_/PtSe_2_ homostructure. In other words, considering the theoretical hot carrier mean free path in the metal (≈10–60 nm)^[^ ^31^ ^]^ and its low resistance (Figure 3c), it is natural for the carrier transport to occur in the in‐plane direction in the PtSe_2_/PtSe_2_ homostructure, just as each film is connected in parallel. On the other hand, we observed the effect of the *S*_int_ in the vertically stacked PtSe_2_/PtSe_2_ homostructure (*N* = 2) as shown in Figure 3c,d, and Figure S3 (Supporting Information).

Since the *S*_int_ could not be observed in the PtSe_2_/PtSe_2_ homostructure composed of the 10‐nm‐thick PtSe_2_ thin film (Figure 4a,b), we selected the PtSe_2_ thin film with a thickness of 3 nm and alternatingly piling the same PtSe_2_ film to fabricate vertically stacked PtSe_2_/PtSe_2_ homostructures with up to eight layers (*N* = 1 – 8) by a wet‐transfer method (Figure 1a) to observe the *S*_int_ induced at the interface between the PtSe_2_ films in the vertically stacked PtSe_2_/PtSe_2_ homostructures. In Figure 4c, we observed that the *S*_||_ value for PtSe_2_/PtSe_2_ homostructure (*N* = 2) increased to 120% compared to that of the PtSe_2_ thin film (3 nm), implying that the *S*_int_ was added to the *S*_||_ of the PtSe_2_ thin film (3 nm).^[^ ^23^ ^]^ When comparing the other PtSe_2_/PtSe_2_ homostructure using the 10 nm thick PtSe_2_ thin film (Figure 4a,b), we found that the thickness of each film that made up the homostructure plays an important role in adding the *S*_int_ component in the carrier transport under the transverse temperature gradient. On the other hand, the measured *R*‐value for the PtSe_2_/PtSe_2_ homostructure (*N* = 2) decreases from 500 to 298 kΩ, which also can be explained by the conventional composite resistance model in two parallel circuits.

As mentioned above, the *S*_int_ is the interface‐induced effect, thus we measured the TE properties as the number of the PtSe_2_ films (each thickness of 3 nm) increased up to eight (Figures 4e–h and Figure S4, Supporting Information). With increasing number of PtSe_2_ films, we observed that the *S*_||_ value of vertically stacked PtSe_2_/PtSe_2_ homostructures, consisted of the 3‐nm‐thick PtSe_2_ thin film, increases linearly to a maximum of 188 µV K^−1^ for four layers (*N* = 4) and then decreases as *N* exceeded 4. The *S*_||_ value of the PtSe_2_/PtSe_2_ homostructure (*N* = 4) was 261% more than the value for the parallel conductor model (Figure 4e), while the measured *R*‐value followed the parallel conductor model (Figure 4f). In addition, the in‐plane electrical conductivities (*σ*_||_) of the stacked PtSe_2_/PtSe_2_ homostructures at 300 K were determined to be in the range from ≈8.2 to 1–3.3 S cm^−1^ (Figure 4g), implying that the *σ*_||_ value remains nearly the same regardless of the number of stacking PtSe_2_ films. As a result, the calculated in‐plane power factor (PF_||_ = *S*_||_^2^ *σ*_||_) of the six‐stacked PtSe_2_/PtSe_2_ homostructure (*N* = 6) is ≈570% more than that for the single 3 nm thick PtSe_2_ film, which can be attributed to the strong decoupling phenomenon between the *S*_||_ and *σ*_||_ due to the formation of interfaces in the stacked PtSe_2_/PtSe_2_ homostructure. This result clearly indicates that the stacked PtSe_2_/PtSe_2_ homostructure is a novel and challenging scheme to break the strong coupling of Seebeck coefficient and electrical conductivity through the vertically stacked homostructures. Figure 4i,j shows the Δ*T*_||_ and the *S*_||_ for the PtSe_2_ thin film (*N* = 1) and vertically stacked PtSe_2_/PtSe_2_ homostructure (*N* = 6) as functions of the measurement time in CAU‐SYS (Figure S5, Supporting Information), where we can identify the driving force that contributed to the increased *S*_||_ value of the stacked PtSe_2_/PtSe_2_ homostructure. For both PtSe_2_ thin film (*N* = 1) and vertically stacked PtSe_2_/PtSe_2_ homostructure (*N* = 6), we found that the measured Δ*T*_||_ were well maintained during the *S*_||_ measurement in the range of 1–5 K (top images in Figure 4i,j). We observed that there is a large variation especially at the low Δ*T*_||_ = 1−2 K for vertically stacked PtSe_2_/PtSe_2_ homostructure (*N* = 6) compared to that for PtSe_2_ thin film (*N* = 1, bottom images in Figure 4i,j). However, it was observed that the fluctuation in *S*_||_ decreases as the temperature difference Δ*T*_||_ increases up to 5 K for vertically stacked PtSe_2_/PtSe_2_ homostructure (*N* = 6, bottom image in Figure 4j), indicating that the main goal of achieving the large fluctuation in *S*_||_ (Figure 4j) is to increase the *S*_int_. Furthermore, the longitudinal temperature difference (Δ*T*_z_) becomes the driving force to occur the momentum transfer due to the carrier‐interface interaction between lower and upper PtSe_2_ layers, increasing the *S*_||_ in the stacked PtSe_2_/PtSe_2_ homostructures.

**Figure 5:**
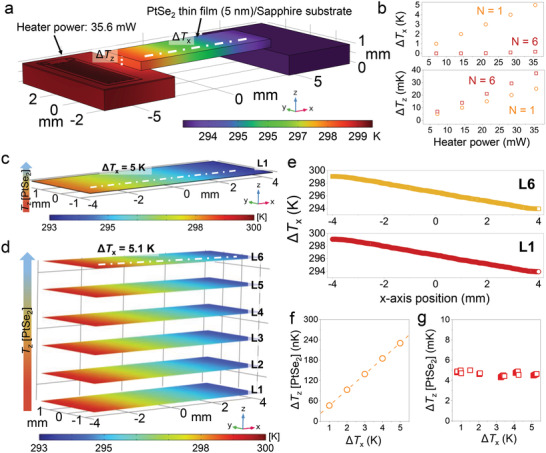
COMSOL simulation results in the 5‐nm‐thick PtSe_2_ thin film (*N* = 1) and the vertically stacked PtSe_2_/PtSe_2_ homostructure (*N* = 6). a) Calculated temperature distribution when measuring the Seebeck coefficient of the 5 nm thick PtSe_2_ thin film// sapphire substrate using the CAU‐SYS. The Δ*T*_x_ were obtained from the temperature difference between (−4, −1) and (4, −1) at the surface of PtSe_2_ thin film ((*x*, *y*) refers to the *x* and *y* positions on the PtSe_2_ thin film). The Δ*T*_z_ was calculated from the edge position (*x* = −4) along the *z*‐axis direction. b) The Δ*T*_x_ and Δ*T*_z_ values obtained for the PtSe_2_ thin film (*N* = 1) and vertically stacked PtSe_2_/PtSe_2_ homostructure (*N* = 6), respectively. The transverse temperature difference of each layer of c) the PtSe_2_ thin film (*N* = 1) and d) vertically stacked PtSe_2_/PtSe_2_ homostructure (*N* = 6) at Δ*T*_x_ ≈ 5 K. L1–L6: the number of PtSe_2_ layer on the sapphire substrate. e) Comparison of the transverse temperature distributions of L1 and L6 in the PtSe_2_ homostructure. The obtained Δ*T*_z_ PtSe_2_ thin film (*N* = 1) and vertically stacked PtSe_2_/PtSe_2_ homostructure (*N* = 6) at Δ*T*_x_ ≈ 5 K.

Through a finite element method (FEM) simulation, we can observe these features more clearly. Thus, we conducted the finite‐element method using COMSOL Multiphysics simulator (version 6.0) with the heat transfer module (**Figure** **5**a–g and Figures S6–S9, Supporting Information) to ensure the contribution of Δ*T*_||_ in the carrier transport in vertically stacked PtSe_2_/PtSe_2_ homostructures. In this calculation, we set conditions similar to that of the experiment to investigate the temperature distribution in the transverse and longitudinal directions according to the interface formation. With a PtSe_2_ thin film (*N* = 1), we adopted a 5 nm thick PtSe_2_ layer on the sapphire substrate with a thickness of ≈430 µm (Figure 5a). The detailed parameters are listed in Table S1 (Supporting Information). Δ*T*_||_ was obtained by the in‐plane (transverse) temperature difference (Δ*T*_x_) on the surface of PtSe_2_ thin film (Δ*T*_x_) (Figure 5a) using two probes with a distance of 7 mm in the COMSOL Multiphysics. Simultaneously, we also obtained the out‐of‐plane (longitudinal) temperature difference (Δ*T*_z_) in the PtSe_2_ thin films on the sapphire substrate (Figure 5a) at the edge of the samples located near the strain gauge heater. For a 5 nm thick PtSe_2_ thin film (*N* = 1), Δ*T*_x_ was increased linearly with increasing heater power (Figure 5b). When evaluating the *S*_||_ value of the vertically stacked PtSe_2_/PtSe_2_ homostructure (*N* = 6), if the heater power is set at the same value as that of PtSe_2_ thin film (*N* = 1), the calculated Δ*T*_x_ of the vertically stacked PtSe_2_/PtSe_2_ homostructure (*N* = 6) is considerably smaller than that of the single PtSe_2_ thin film. Therefore, we performed the calculations by setting the same condition of Δ*T*_x_ ≈ 5 K on the surface of each PtSe_2_ film and the vertically stacked PtSe_2_/PtSe_2_ homostructure up to *N* = 6 (Figure 5c,d). We further confirmed that the transverse temperature distribution on each film of the PtSe_2_/PtSe_2_ homostructure (*N* = 6) remained the same at Δ*T*_x_ = 5.1 K (Figure 5e). In Figures 5f and g, we compared the Δ*T*_z_ distribution as a function of the Δ*T*_x_ in the range of 1–5 K for both PtSe_2_ thin film (*N* = 1) and vertically stacked PtSe_2_/PtSe_2_ homostructure (*N* = 6). We found the Δ*T*_z_ for the vertically stacked PtSe_2_/PtSe_2_ homostructure (*N* = 6) was determined as ≈5 mK at the Δ*T*_x_ = 5.1 K, while it is ≈0.2 µK for the PtSe_2_ thin film (*N* = 1). This considerably large Δ*T*_z_ in the vertically stacked PtSe_2_/PtSe_2_ homostructure (*N* = 6) is sufficient to act as a driving force in the carrier transport when applying the transverse temperature gradient along the sample. A more detailed explanation of the relationship between the carrier transport in the stacked PtSe_2_/PtSe_2_ homostructure is discussed in the section below.

**Figure 6:**
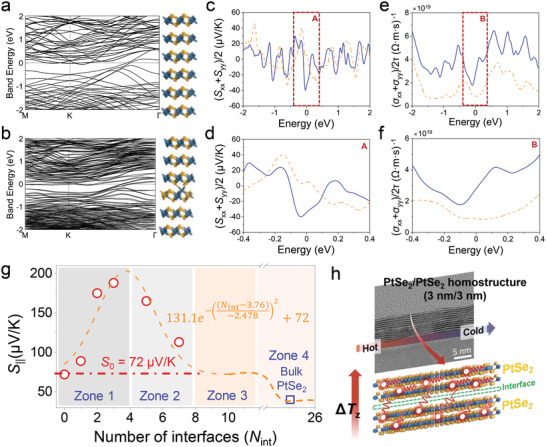
DFT calculation and mechanism of the in‐plane Seebeck coefficient for the alternatingly stacked PtSe_2_/PtSe_2_ homostructure (3 nm/3 nm) with interfacial number. The calculated electronic band structure and atomic structure for the (2 × 2) six layers of PtSe_2_ a) without and b) with defects. The defects were generated a Pt‐Se exchange for comparison. In terms of the atomic structure, the blue and yellow balls represent the Pt and Se atoms, respectively. c) The calculated in‐plane Seebeck tensor for the six layers of PtSe_2_ without (blue solid) and with defects (orange dot), respectively. d) Enlarged image marked area in (c). e) The calculated in‐plane electrical conductivity tensor divided to the relaxation time for the six layers of PtSe_2_ without (blue solid) and with defects (orange dot), respectively. f) Enlarged image marked area in (e). g) The distribution of *S*_||_ for the vertically stacked PtSe_2_/PtSe_2_ homostructure is divided into four zones, depending on the number of interfaces. Curve fitting results using our experimental data using an arbitrary function based on the Gaussian function. After the peak value, the *S*_||_ of the vertically stacked PtSe_2_/PtSe_2_ homostructure, consisted of the 3 nm thick PtSe_2_ thin film, decreased due to the carrier mean free path and interface formation between the PtSe_2_ layers. h) Schematic of the *S*_int_ through momentum transfer in the vertically stacked PtSe_2_/PtSe_2_ homostructure (3 nm/3 nm) under the transverse temperature gradient.

Furthermore, we conducted additional density functional theory (DFT) calculations on the defect at the interface (**Figure** **6**a−f). The in‐plane lattice parameters were fixed at the lattice parameters of bulk PtSe_2_. The planar directions (2 × 2) unit cell was used, and a vacuum space larger than 46 Å was added in the direction normal to the PtSe_2_ surface. The Seebeck tensor calculations were performed for six layers of PtSe_2_, and six layers of PtSe_2_ with defects composed of a Pt‐Se exchange were used for comparison. In Figure 6c−d, the average in‐plane Seebeck tensor components are shown. The overall magnitude of the in‐plane Seebeck tensor component of six layers of PtSe_2_ with defects is larger than that without defects. The electronic band structures consisted of six layers of PtSe_2_ with and without defects were also computed (Figure 6a−b). The metallic band structure is maintained with the introduction of defects. However, some valence and conduction bands are pushed toward the Fermi level and become less dispersive with defects, which cause the enhanced Seebeck effect. According to the Figure 6a–f and Figure S10 (Supporting Information), it can be confirmed again that an interface morphology (Figure 2) different from a general vdW interface is one of the factors generating the *S*_int_.

Based on our experimental and calculation results, we concluded that the *S*_int_ in vertically stacked PtSe_2_/PtSe_2_ homostructure is attributed to a number of factors; the thickness of PtSe_2_ thin film constituting PtSe_2_/PtSe_2_ homostructure, out‐of‐plane temperature difference (driving force), and the abnormal interface morphology between PtSe_2_ films. Because each PtSe_2_ film was separated by the interface, the *S*_int_ is occurred by the carrier‐interface interaction. In other words, a momentum transfer of electrical carriers from the lower PtSe_2_ film to the upper PtSe_2_ film increases the *S*_||_ without affecting the *σ* as like the phonon drag effect.^[^ ^32^, ^33^ ^]^ Due to the *S*_int_ is caused by the momentum transfer, the *S*_int_ should be treated independently when considering the total in‐plane Seebeck coefficient of PtSe_2_/PtSe_2_ homostructure, *S*_||_ = $S_{∥}^{PtSe_{2}}$ + S_int_, where $S_{∥}^{PtSe_{2}}$ is the conventional Seebeck coefficient of the PtSe_2_ thin film (*N* = 1).^[^ ^32^, ^33^ ^]^ If the momentum transfer of the carriers was happened by the out‐of‐plane temperature difference, we can expect some quantitative changes in the PtSe_2_/PtSe_2_ homostructure. To confirm this assumption, we calculated the Seebeck effective mass ($m_{s}^{*})$of PtSe_2_ films using a model presented by Snyder^[^ ^34^ ^]^ because the magnitude is proportional to the its effective mass according to the Mott relation.^[^ ^35^ ^]^ The Seebeck effective mass of PtSe_2_ films is determined by the following equation^[^ ^34^ ^]^

(3) $$\begin{array}{ccc} & & \frac{m_{s}^{*}}{m_{e}}=0.924\left(\frac{300K}{T}\right){\left(\frac{n_{H}}{10^{20}{\mathrm{cm}}^{-3}}\right)}^{2/3}\\ & & \times \left[\frac{3{\left(\exp\left[\frac{\left|S\right|}{k_{B}/e}\right]-0.17\right)}^{2/3}}{1+\exp\left[-5\left(\frac{\left|S\right|}{k_{B}/e}-\frac{k_{B}/e}{\left|S\right|}\right)\right]}+\frac{\frac{\left|S\right|}{k_{B}/e}}{1+\exp\left[5\left(\frac{\left|S\right|}{k_{B}/e}-\frac{k_{B}/e}{\left|S\right|}\right)\right]}\right] \end{array}$$

where $m_{s}^{*}$ is the Seebeck effective mass, *m_e_* is the carrier effective mass, *n*_H_ is the charge carrier concentration measured by the Hall effect (*n*_H_ = 1/*eR*_H_, *R*_H_ is Hall resistance) in 10^20^ cm^−3^, *T* is the absolute temperature in K, and *k*_B_/e = 86.3 µV K^−1^. Through our Hall measurement data (Table S2, Supporting Information), we calculated the $m_{s}^{*}$ for the PtSe_2_ thin film (3 nm, *N* = 1) and PtSe_2_/PtSe_2_ homostructures (3 nm thick PtSe_2_ thin film, *N* = 2 – 4) as shown in Table S3 (Supporting Information). As a result, we found out that the calculated $m_{s}^{*}$ of the PtSe_2_/PtSe_2_ homostructure (*N* = 4) is ≈3 times larger than that of the PtSe_2_ thin film (*N* = 1).

The behavior of *S*_||_ in the vertically stacked PtSe_2_/PtSe_2_ homostructures (3‐nm‐thick PtSe_2_ thin film, *N* = 1 – 8) with increasing number of interfaces or interface layers can be explained by dividing it into four zones (Figure 6g). First, in Zone 1, the *S*_||_ linearly increases by increasing the PtSe_2_ film up to *N* = 4. This can be explained by the *S*_int_ induced at the interface between the 2D/2D PtSe_2_ films, resulting in the highest *S*_||_ exceeding ≈188 µV K^−1^ at 300 K in the PtSe_2_/PtSe_2_ homostructure (*N* = 4, Figure 6h). As we discussed in the previous section of the COMSOL result (Figure 5), we observed that the Δ*T*_z_ of the six‐stacked PtSe_2_/PtSe_2_ homostructure acts as a driving force to make an interaction between carriers and interface in the lower PtSe_2_ layer upward, in turn enhancing the overall *S*_||_ in the stacked PtSe_2_ structure. As a result, the *S*_||_ value increases linearly as the number of stacking PtSe_2_ film increases (Figure 4e), while the *σ*_||_ value of the stacked PtSe_2_/PtSe_2_ homostructure remains unchanged with increasing stacking number (Figure 4g). In the carrier transport mechanism, the interface space between the PtSe_2_ layers becomes an effective conducting channel when the temperature gradient is applied along the samples (upper image in Figure 6h). Accordingly, the interface between 2D/2D layers plays an important role in occurring the momentum transfer produced at the interface in the longitudinal direction when measuring the transverse Seebeck coefficient of the vertically stacked PtSe_2_/PtSe_2_ homostructures. On the other hand, in Zone 2, the *S*_||_ value decreases as the film number increases. This result is attributed to the short mean free path (MFP) of PtSe_2_ films and increased carrier scattering at the interface with an increased number of the PtSe_2_ film in vertically stacked PtSe_2_/PtSe_2_ homostructures. Based on the results of a previous study,^[^ ^36^ ^]^ the theoretical MFP of hot electrons in metals was reported as 10–60 nm. In addition, the electronic MFP of 2D bulk MoS_2_ material was ≈14 nm at room temperature.^[^ ^37^ ^]^ Accordingly, the *S*_||_ value measured on the top PtSe_2_ film (Figure 1b,c) tends to decrease when the total thickness of stacked PtSe_2_ layers exceeded the MFP of the PtSe_2_ film (>14 nm) because the hot carriers are not moved from the lower PtSe_2_ film through the interface when the temperature gradient is applied along the samples. In Zone 3, with an increasing number of films (Figure 6g), we noticed that the *S*_||_ value of the stacked PtSe_2_/PtSe_2_ homostructures was the same as that of the single PtSe_2_ film regardless of the number of stacked PtSe_2_ films, confirming that the normal parallel conduction model is applicable to the stacked PtSe_2_/PtSe_2_ homostructures (*N* ≥ 8). Finally, in Zone 4, when the thickness of the stacked PtSe_2_ film increases to >1 µm, the *S*_||_ value becomes the same as that (≈40 µV/K) of the bulk PtSe_2_ (Figure 6g).^[^ ^38^ ^]^ In Figure 6g, the *S*_||_ trends to follow the equation, $S_{∥}=131.1e^{-{\left(\frac{\left(N_{\mathrm{int}}-3.76\right)}{-2478}\right)}^{2}}+S_{0}$, where *N*_int_ is the number of interfaces (*N*_int_ = *N* − 1) and *S*_0_ is the in‐plane Seebeck coefficient of the single PtSe_2_ (∼3 nm) film.

## 3 Conclusion

In this study, we fabricated millimeter‐scale 2D homostructures using PtSe_2_ thin films with various thicknesses. An abnormally high in‐plane Seebeck coefficient was observed, which was attributed to the extra interfacial Seebeck effect due to the momentum transfer when occurring carrier‐interface interaction by longitudinal temperature difference under a transverse temperature gradient along the samples, after forming a vertically stacked PtSe_2_/PtSe_2_ homostructure. Particularly, the stacked PtSe_2_/PtSe_2_ homostructure consisted of the 3 nm thick PtSe_2_ films and the in‐plane Seebeck coefficient and electrical conductivity was decoupled, enhancing the in‐plane power factor in stacked PtSe_2_/PtSe_2_ homostructures due to the interfacial formation, MFP, and momentum transfer at the abnormal interfaces between PtSe_2_ films.

## 4 Experimental Section

### Large‐Area Multilayer PtSe_2_ Film Growth

A simple selenization procedure using H_2_ and N_2_ gases in a low‐pressure chemical vapor deposition (CVD) procedure to synthesize PtSe_2_ thin films was followed. First, Pt thin films were deposited on a SiO_2_/Si substrate (thickness of SiO_2_ is ≈270 nm) via radio‐frequency magnetron sputtering in Ar atmosphere. Substrates were ultrasonically cleaned with acetone and isopropyl alcohol, consecutively, for 10 min before Pt deposition. The Pt deposition was performed with an RF power of 16 W and a working Ar pressure of 2.0 × 10^−3^ Torr at room temperature. Under the background pressure of 8.0 × 10^−5^ Torr, CVD furnace temperature was then raised to 400−500 °C for selenization and maintained for 30 min under 5% H_2_ flow in Ar.

### Wet Transfer Process to Form Vertically Stacked PtSe_2_/PtSe_2_ Homostructure onto Sapphire Substrates

A wet transfer technique to fabricate the PtSe_2_ homostructure onto the sapphire substrate was employed. A polymethylmethacrylate (PMMA) solution was prepared using toluene as solvent and was spun onto the PtSe_2_ thin films. Subsequently, PMMA‐coated multilayer PtSe_2_ films were immersed in the hydrofluoric solution (HF, 0.5 wt.%) for etching at room temperature for 1 min. The PMMA‐coated multilayer PtSe_2_ films were detached from the substrate and rinsed with deionized water several times. One hundred clean sapphire substrates (thickness ≈430 µm) were used to retrieve the floating PMMA‐coated PtSe_2_ films. After heating at 110 °C for 30 min to remove the moisture remaining on the surface, PMMA‐coated PtSe_2_/sapphire ensembles were dipped into an acetone bath to remove the PMMA. To prepare PtSe_2_ homostructure, we repeated the same process mentioned above.

### In‐Plane Seebeck Coefficient Measurements for PtSe_2_ Films and Vertically Stacked PtSe_2_/PtSe_2_ Homostructures on Sapphire Substrates

To measure the in‐plane TE properties of the PtSe_2_ thin films, the PtSe_2_ films to the sapphire substrate to avoid the substrate effect during the Seebeck coefficient measurement was transferred. In the measurement system (i.e., in‐house CAU‐SYS), the transverse temperature difference was applied with a strain gauge from left to right, which is parallel to the surface of PtSe_2_ thin film. In this study, the lateral dimensions of the PtSe_2_ thin films at 3 mm × 6 mm (width × length) was fixed. a strain gauge of 120 Ω and two T‐type thermocouples in the heater that simultaneously monitored and controlled the temperature difference located to the surface of the PtSe_2_ thin film was used. Applying an electric current to the strain gauge generated a temperature gradient Δ*T* across the samples. Δ*T* was measured using the two T‐type thermocouples. Moreover, the in‐plane Seebeck voltages Δ*V* using tungsten needles between sample end positions, with conductive Ag paste to minimize electrical contact resistivity between the needles and PtSe_2_ thin films was measured.^[^ ^39^, ^40^ ^]^ In‐plane Seebeck coefficients for PtSe_2_ thin films for each Δ*T* were subsequently calculated by linearly fitting Δ*V* on Δ*T* using *S* = − Δ*V*/Δ*T*. All measured data were monitored and controlled using a CompactDAQ with LabVIEW software (National Instruments, USA).

### COMSOL Multiphysics Simulation

To support our measurement data, the COMSOL simulation similar to the experimental setup was conducted. The lateral dimensions of the sample were 3 mm × 6 mm (width × length) and the thermal grease (Arctic MX‐4) component was applied between the Cu block and the sapphire substrate. Since the in‐plane temperature difference was applied with a strain gauge from left to right in our CAU‐SYS, the temperature difference along the *x*‐axis (Δ*T*_x_) was controlled by the applied heater power. Detailed material parameters are listed in Table S1 (Supporting Information).

### First‐Principle Calculations for PtSe_2_

The calculations were performed using the ab‐initio total‐energy and molecular‐dynamics program, called the Vienna ab‐initio simulation program (VASP), developed at the Institut für Materialphysik of the Universität Wien, using the projector‐augmented‐wave (PAW) approach^[^ ^41^ ^]^ and Perdew–Burke–Ernzerhof (PBE) generalized gradient approximation.^[^ ^42^ ^]^ The cutoff energy of 250 eV was used for a plane‐wave basis set. The maximum remaining force on each atom was less than ≈0.015 eV Å^−1^ for structural relaxation. The Monkhorst‐Pack scheme was used to sample the Brillouin zone. The k‐point meshes for the structural relaxation and self‐consistent charge density are 7 × 7 × 1. The average in‐plane Seebeck tensor component (*S*_xx_ + *S*_yy_)/2 was calculated using the BoltzTraP code.^[^ ^43^, ^44^ ^]^ The k‐point meshes for the transport tensor calculations are 19 × 19 × 1.

## Conflict of Interest

The authors declare no conflict of interest.

## Supporting information

Supporting Information

## Data Availability

The data that support the findings of this study are available in the supplementary material of this article.
