# Supplementary material for: Abnormal Seebeck Effect in Vertically Stacked 2D/2D PtSe2/PtSe2 Homostructure
Source: Adv Sci (Weinh). 2022 Nov 10;9(36):2203455. doi: 10.1002/advs.202203455 (PMC9799017; doi:10.1002/advs.202203455)
Supplement: Supplementary file 1 — Supporting Information [file ADVS-9-2203455-s001.pdf]

ADVANCED SCIENCE
Open Access

Supporting Information

for *Adv. Sci.*, DOI 10.1002/advs.202203455

Abnormal Seebeck Effect in Vertically Stacked 2D/2D $PtSe_2/PtSe_2$ Homostructure

*Won-Yong Lee, Min-Sung Kang, Jae Won Choi, Si-Hoo Kim, No-Won Park, Gil-Sung Kim, Yun-Ho Kim, Eiji Saitoh, Young-Gui Yoon and Sang-Kwon Lee**

Supporting Information

Abnormal Seebeck effect in vertically stacked 2D/2D $PtSe_2/PtSe_2$ homostructure

Won-Yong Lee[1,2,†], Min-Sung Kang[1, †], Jae Won Choi[1, †], Si-Hoo Kim[1], No-Won Park[1], Gil-Sung Kim[1], Yun-Ho Kim[1], Eiji Saitoh[3], Young-Gui Yoon[1], Sang-Kwon Lee[1,*]

[1]Department of Physics, Center for Berry Curvature based New Phenomena, Chung-Ang University, Seoul 06974, Republic of Korea
[2]Division of Solid State Electronics, Department of Electrical Engineering, Uppsala University, Lägerhyddsvägen 1, Uppsala 75103, Sweden
[3]Department of Applied Physics, The University of Tokyo, Tokyo 113-8656, Japan

*Corresponding author. Email: sangkwonlee@cau.ac.kr
[†]These authors contributed equally to this work

Table S1. Material parameters for COMSOL calculations.

Material	Density [kg/m^3]	Heat capacity at constant pressure [C_p, J/kg·K]	Thermal conductivity [W/m·K]
Sapphire substrate (Al_2O_3)	3,980 [1]	779.15*	40 [1]
$PtSe_2$ (5-nm)	9,540 [2]	206.49 [3]	1.08 (‖)/0.12 (⊥) [2]
Copper	8,960*	348.8*	392.81*
Bakelite	1,300*	920*	0.2*
Gold	19,276*	128.78*	317.32*
Kapton H	1,420*	758*	0.81*
Thermal grease	2,600*	1,200*	8.5 [4]

*These values are taken from COMSOL Material library.

†The interfacial thermal resistance (ITR) between $PtSe_2$ (thickness of 5 nm) and $PtSe_2$ (thickness of 5 nm) was set to 1×10^{-9} K·m^2/W.

‡The ITR between $PtSe_2$ and Sapphire substrate was determined to be 1×10^{-9} K·m^2/W.

Table S2. Hall measurement results for single $PtSe_2$ film and $PtSe_2/PtSe_2$ homostructures. All measurement data were obtained from the van der Pauw method under a 0.5 T magnetic field. Each $PtSe_2$ layer has a thickness of 3 nm.

Sample	Carrier concentration n [/cm^3]	Carrier mobility μ [cm^2/V·s]	Electrical conductivity [S/cm]
$PtSe_2$ thin film (N = 1)	$5.1\times10^{18} \pm 8.7\times10^{17}$	2.5 ± 0.4	2.04 ± 0.33
$PtSe_2$ homostructure (N = 2)	$2.8\times10^{18} \pm 5.5\times10^{17}$	3.1 ± 0.6	1.39 ± 0.27
$PtSe_2$ homostructure (N = 3)	$3.0\times10^{18} \pm 5.5\times10^{17}$	2.1 ± 0.5	1.01 ± 0.24
$PtSe_2$ homostructure (N = 4)	$3.8\times10^{18} \pm 4.4\times10^{17}$	2.8 ± 0.4	1.70 ± 0.24

Table S3. Calculated Seebeck effective mass (m_s^*) through our Hall measurement data. The second and third column refer to the relative effective mass divided by the carrier effective mass (m_e). The m_e was used the effective mass of electron or the calculated value from the Snyder's model.[5]

Sample	m_s^* [kg]	m_s^*/m_e [m_e = 9.11×10^{-31} kg]	m_s^*/m_e [m_e – obtained from calculation]
$PtSe_2$ thin film (N = 1)	9.7×10^{-32}	0.106	0.098
$PtSe_2$ homostructure (N = 2)	8.6×10^{-32}	0.094	0.086
$PtSe_2$ homostructure (N = 3)	2.0×10^{-31}	0.223	0.205
$PtSe_2$ homostructure (N = 4)	2.9×10^{-31}	0.315	0.289

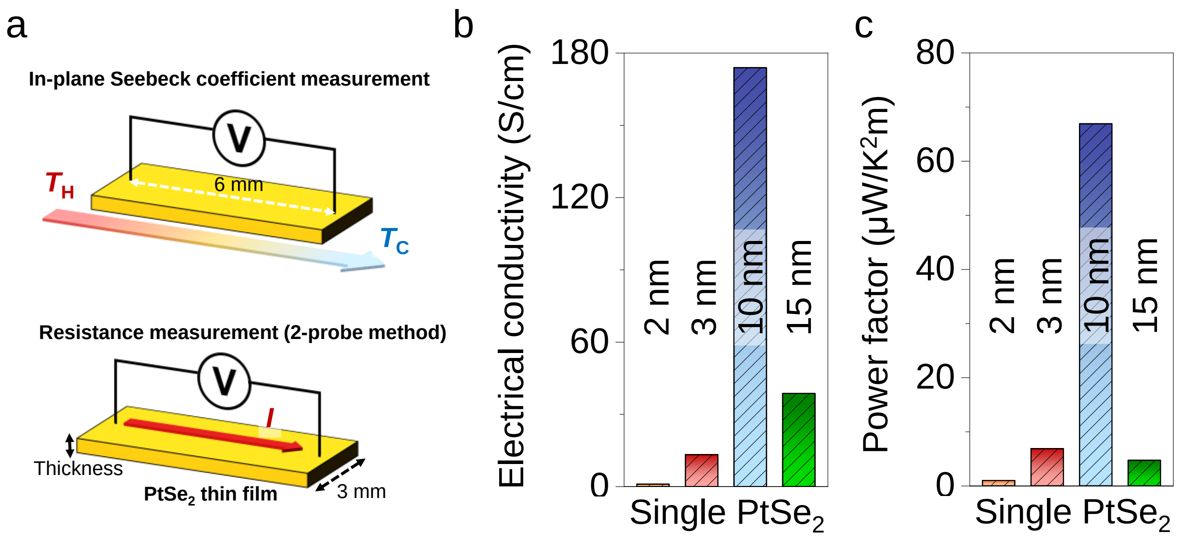

Figure S1. (a) Schematics for measuring the in-plane Seebeck coefficient and electrical resistance of the single $PtSe_2$ thin film using the CAU-SYS. (b) The obtained electrical conductivity of single $PtSe_2$ thin films using the 2-point-probe method. (c) In-plane power factor of single $PtSe_2$ thin films calculated according to the thickness of the $PtSe_2$ thin films.

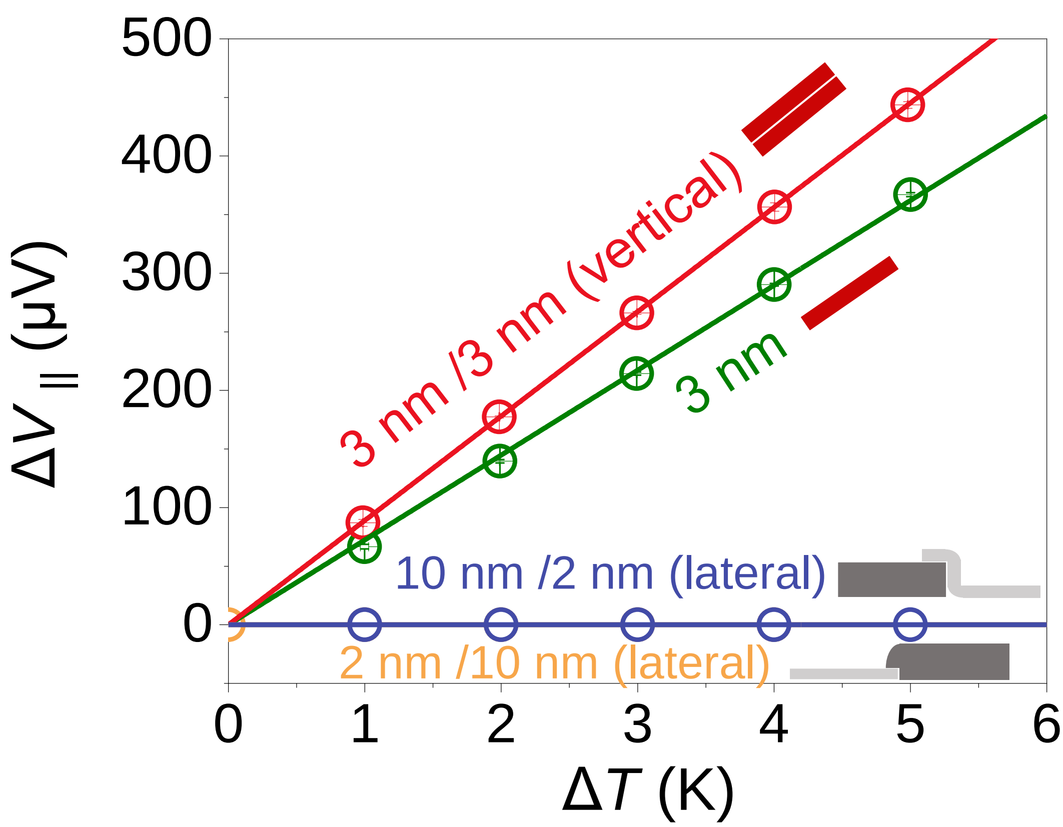

Figure S2. Measured in-plane Seebeck voltage of the single $PtSe_2$ (~3 nm), vertically stacked $PtSe_2/PtSe_2$ bilayer homostructure (~3 nm/~3 nm), and laterally stacked $PtSe_2/PtSe_2$ bilayer homostructure (~10 nm/~2 nm and ~2 nm/~10 nm, respectively) up to ΔT = 5 K at room temperature.

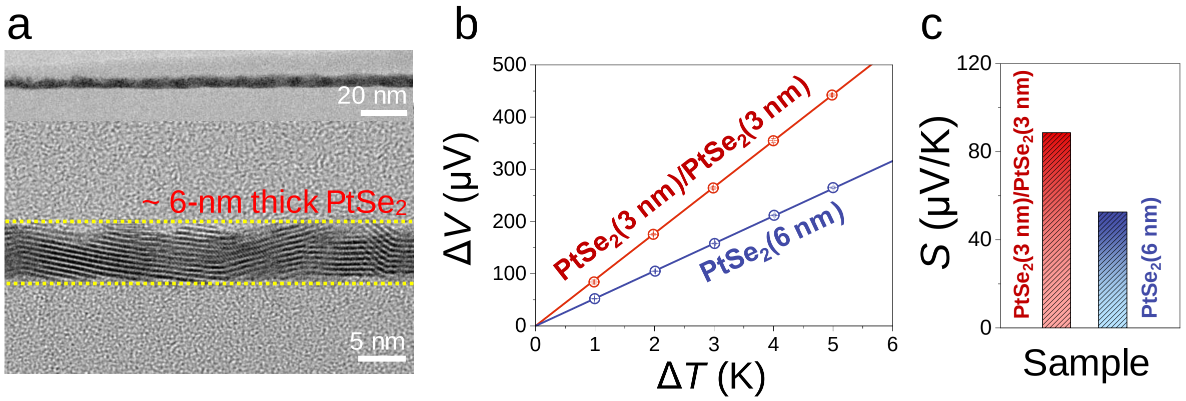

Figure S3. (a) Cross-sectional TEM image of the $PtSe_2$ (6 nm) thin film. In-plane (b) TE voltages and (c) Seebeck coefficients for the $PtSe_2$ (3 nm)/$PtSe_2$ (3 nm), $PtSe_2$ (6 nm) on the sapphire substrate with respect to temperature differences (ΔT) up to 5 K, respectively.

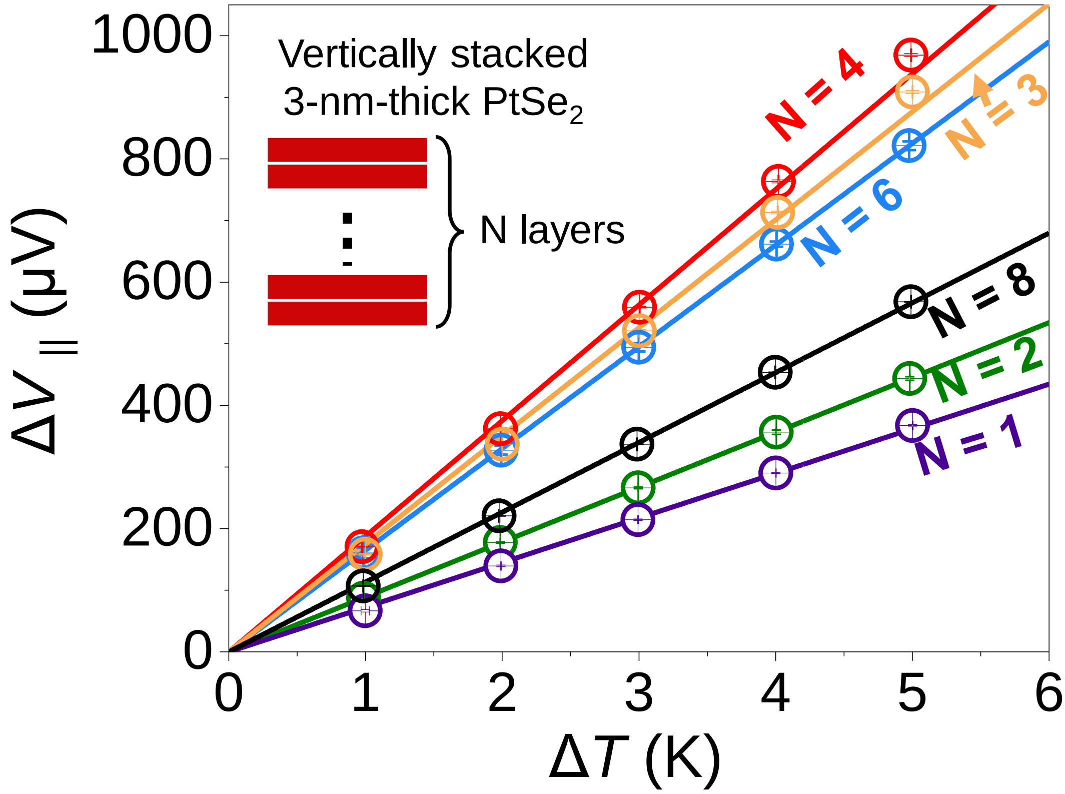

Figure S4. Measured in-plane Seebeck voltage of the stacked $PtSe_2$/$PtSe_2$ homostructures by vertical stacking of single $PtSe_2$ thin films of the same thickness (~3 nm). *N* indicates the number of 3-nm single $PtSe_2$ thin films. We measured the in-plane Seebeck voltage in the Δ*T* range of 1–5 K at room temperature.

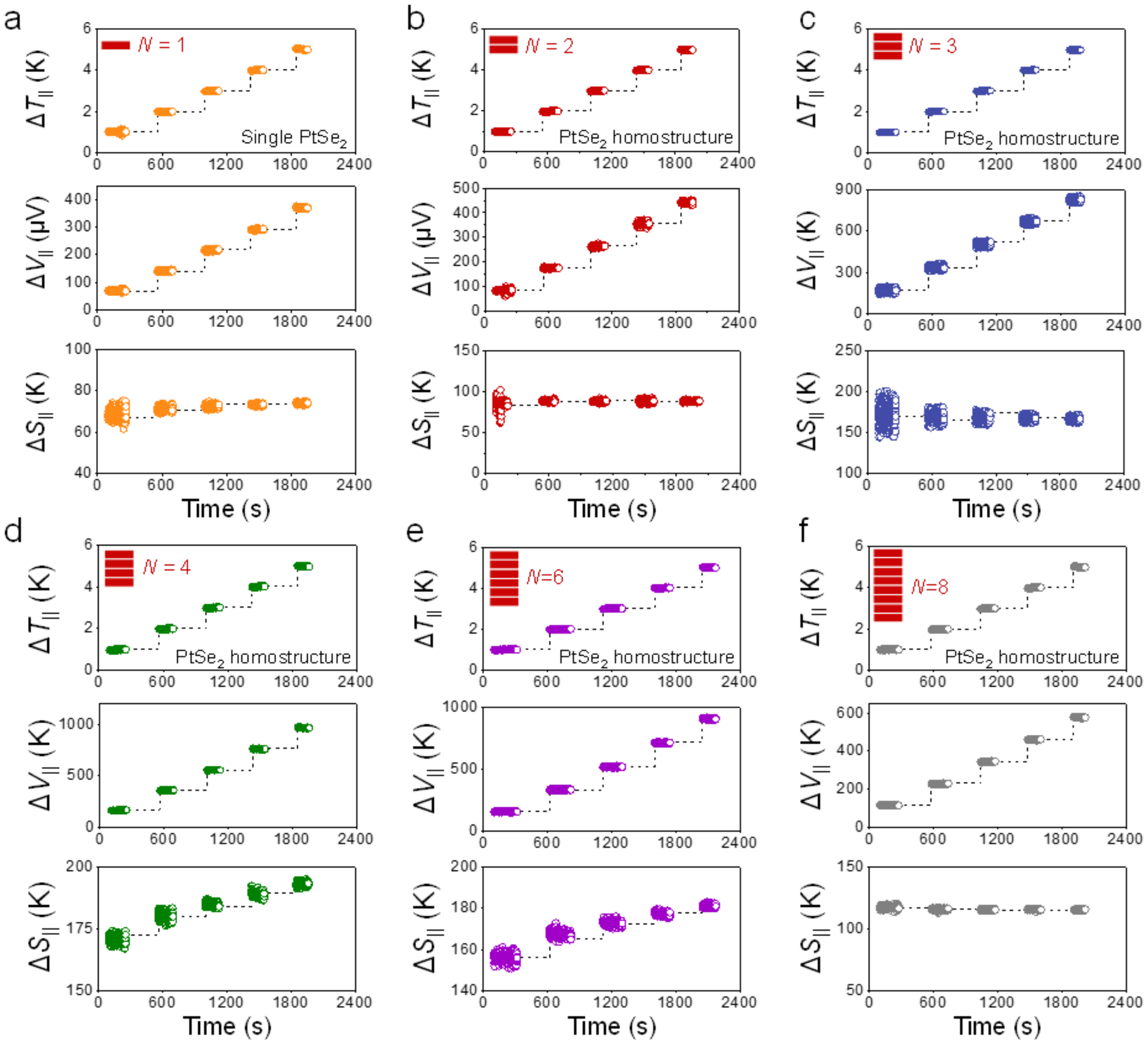

Figure S5. Measured in-plane Seebeck voltages, temperature differences, and obtained Seebeck coefficients at fixed temperature difference for the stacked $PtSe_2/PtSe_2$ (3 nm) homostructures when (a) N = 1, (b) N = 2, (c) N = 3, (d) N = 4, (e) N = 6, and (f) N = 8, respectively.

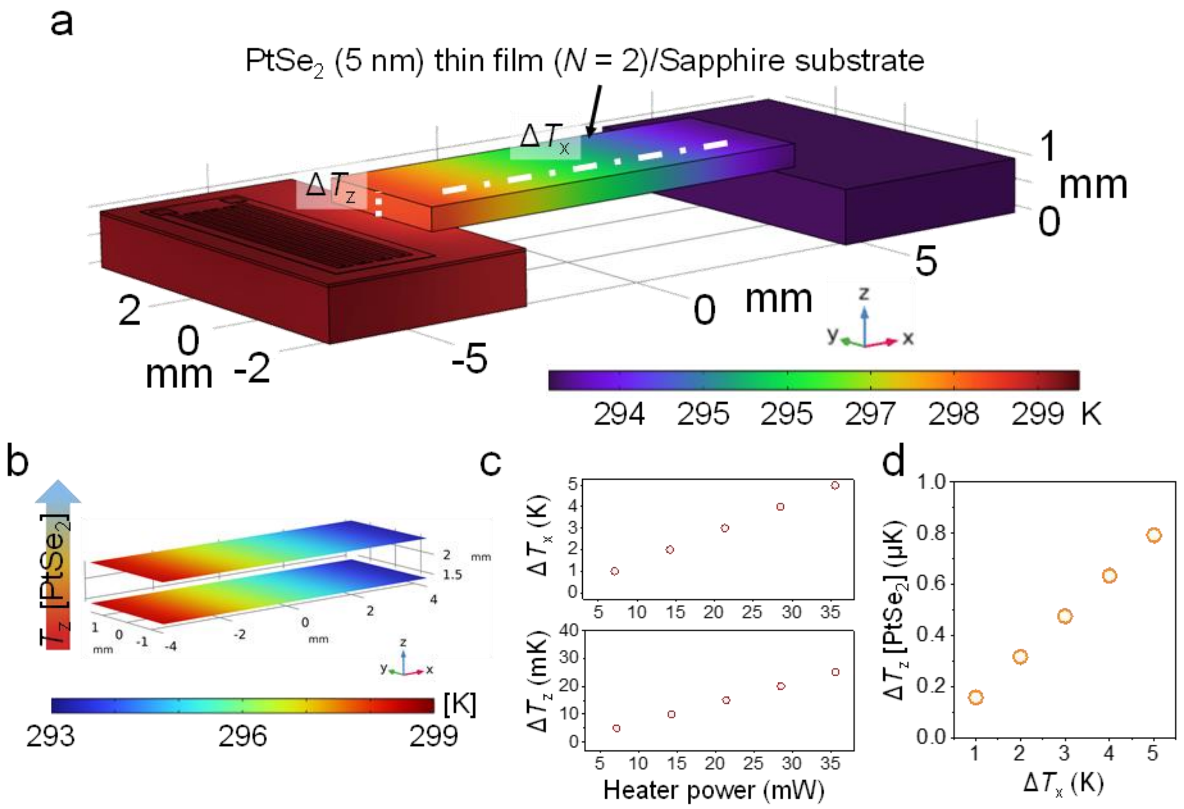

Figure S6. COMSOL simulation results for the two-layer-stacked $PtSe_2/PtSe_2$ homostructure ($N = 2$).

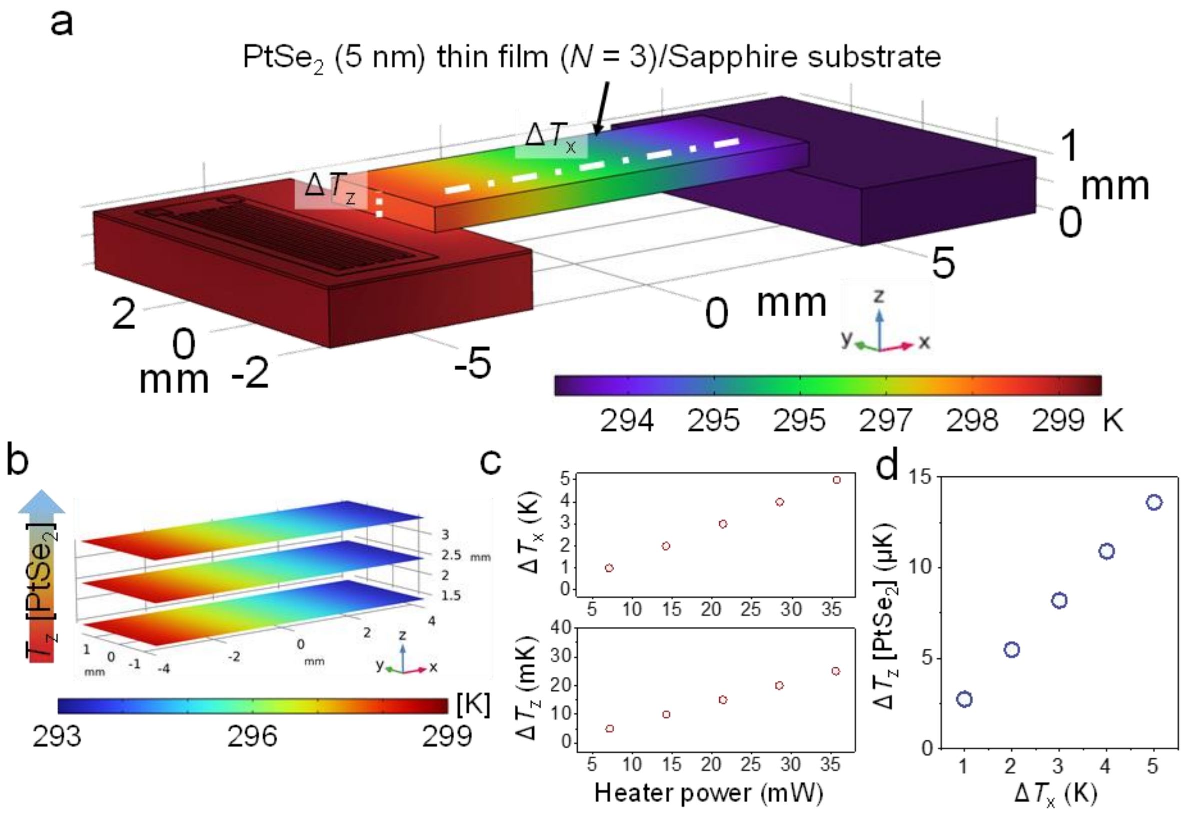

Figure S7. COMSOL simulation results for the three-layer-stacked $PtSe_2/PtSe_2$ homostructure (N = 3).

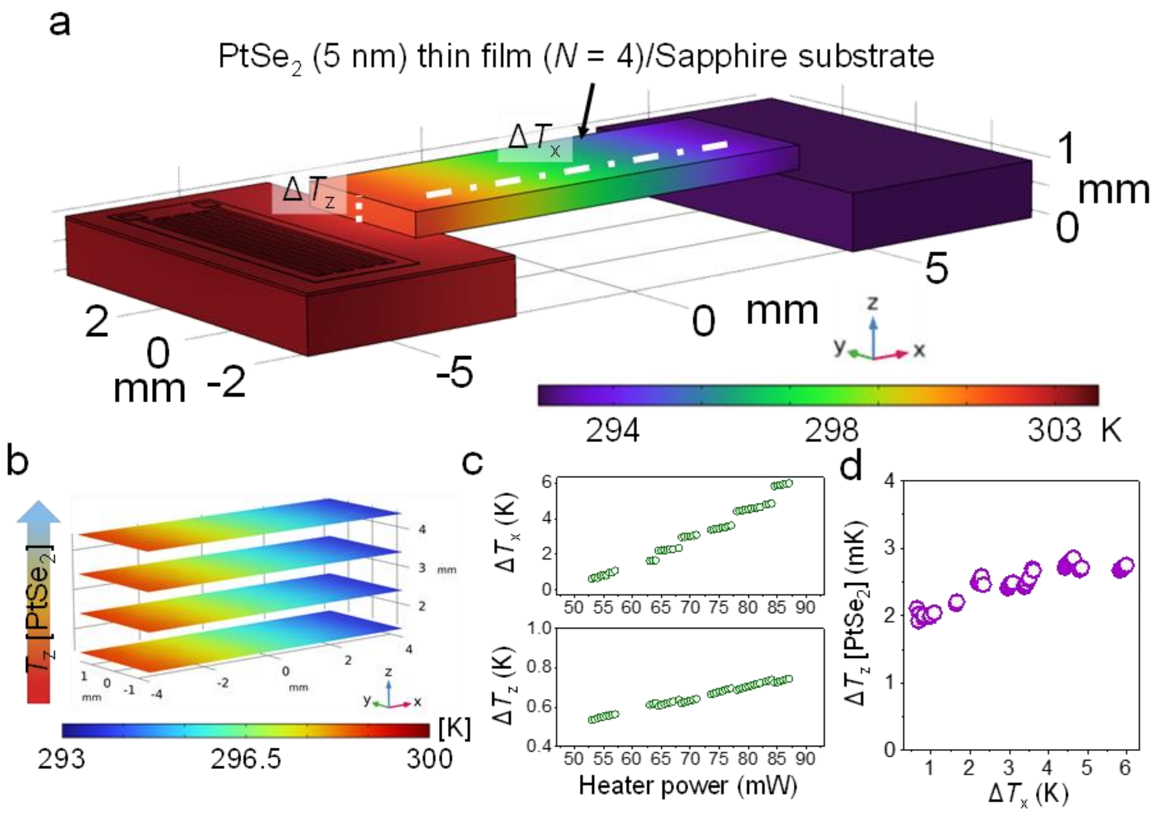

Figure S8. COMSOL simulation results for the four-layer-stacked $PtSe_2$/$PtSe_2$ homostructure (N = 4).

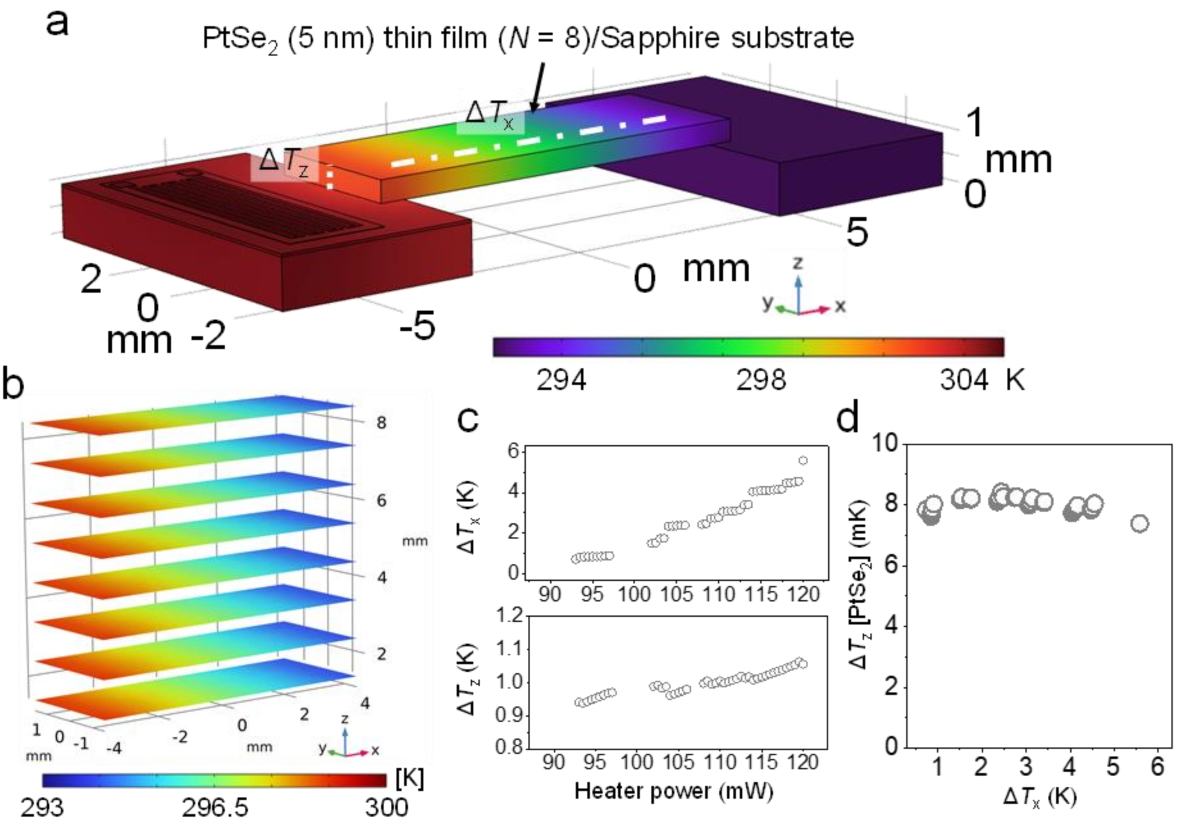

Figure S9. COMSOL simulation results for the eight-layer-stacked $PtSe_2/PtSe_2$ homostructure (N = 8).

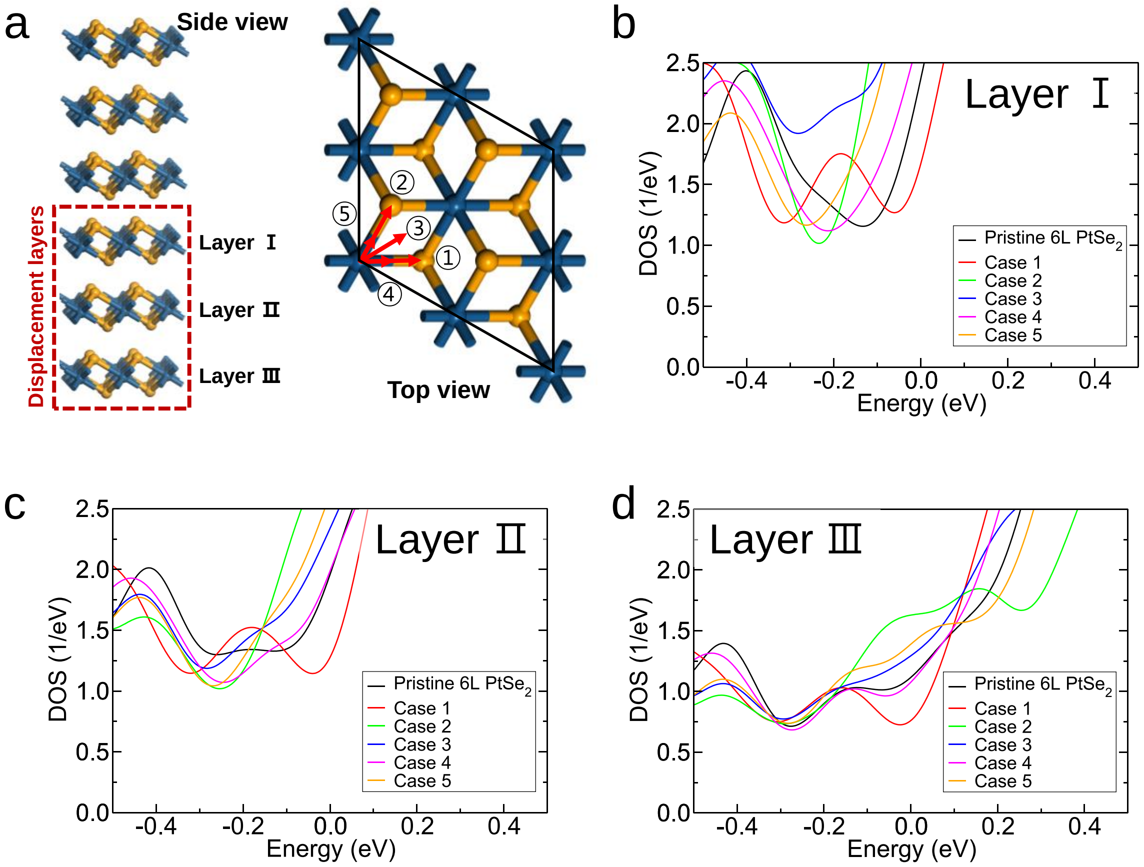

Figure S10. (a) Atomic structure of the 6 layers of $PtSe_2$ in terms of side and top view, respectively. The displaced 3 bottom $PtSe_2$ layers are indexed as I, II, and III. Case 1 – 5 indicate displacements of 3 bottom $PtSe_2$ layers for case 1 – 5, respectively. Projected density of states of the (b) Layer I, (c) Layer II, (d) Layer III, respectively.

References

[1] Vol. 2022.
[2] A. El Sachat, P. Xiao, D. Donadio, F. Bonell, M. Sledzinska, A. Marty, C. Vergnaud, H. Boukari, M. Jamet, G. Arregui, Z. Chen, F. Alzina, C. M. Sotomayor Torres, E. Chavez-Angel, 2021, arXiv:2111.13395.
[3] J.-Q. Lei, K. Liu, S. Huang, X.-L. Zhou, Theoretical Chemistry Accounts 2017, 136, 97.
[4] Vol. 2022.
[5] G. J. Snyder, A. Pereyra, R. Gurunathan, Adv. Funct. Mater. 2022, 32, 2112772.
